# Accuracy of deep learning in diagnosis of apnea syndrome: a systematic review and meta-analysis

**DOI:** 10.3389/fneur.2025.1663851

**Published:** 2025-12-01

**Authors:** Alimila Saiyitijiang, Zhihui Nai, Ying Gao, Ping Fan

**Affiliations:** 1 Cardiac Function Department, Heart Center, The First Affiliated Hospital of Xinjiang Medical University, Ürümqi, China; 2 Department of Science and Technology, Xinjiang Medical University, Ürümqi, China; 3 The Third General Department, The First Affiliated Hospital of Xinjiang Medical University, Ürümqi, China

**Keywords:** deep learning, diagnosis, ECG, meta-analysis, OSAS

## Abstract

**Objectives:**

This systematic review and meta-analysis was carried out to elucidate the accuracy of image-based deep learning (DL) methods in the real-time detection of obstructive sleep apnea syndrome (OSAS).

**Methods:**

A systematic search was conducted for studies published since database establishment up to September 25, 2025, across databases including PubMed, Embase, Web of Science, and the Cochrane Library. The included studies were assessed for risk of bias by using the QUADAS-2 tool. During this meta-analysis, a bivariate mixed-effects model was employed and only synthesized the results from the meta-analysis of the validation sets. Meanwhile, subgroup analyses were conducted based on the generation methods of the validation sets.

**Results:**

A total of 39 original studies were ultimately included, all of which constructed DL images derived from electrocardiogram (ECG) images. Our meta-analysis results suggested that for the comprehensive validation set, the sensitivity, specificity, diagnostic odds ratio (DOR), and the area under summary receiver operating characteristic (SROC) curve were 0.93 (95% CI: 0.90–0.96), 0.95 (95% CI: 0.92–0.96), 252 (95% CI:116–549), and 0.98 (95% CI: 0.42–1.00), respectively. For the independent validation set, the sensitivity, specificity, and SROC curve were 0.93 (95% CI: 0.88–0.96), 0.95 (95% CI: 0.92–0.97), and 0.98 (95% CI: 0.42–1.00), respectively. For the K-fold cross-validation set, the sensitivity, specificity, positive likelihood ratio (LR), and SROC curve were 0.94 (95% CI: 0.88–0.97), 0.94 (95% CI: 0.89–0.96), 15.0 (95% CI: 8.1–27.6) and 0.98 (95% CI: 0.65–1.00), respectively.

**Conclusion:**

The ECGs-based DL models demonstrate ideal accuracy for the detection of OSAS and appear to be a viable method for real-time detection. During our research process, we found that the modeling was actually based on extracting studies from segments of ECGs, but the extracted segments appeared to vary in duration. Since this aspect was not subjected to subgroup analysis in our study, we plan to conduct further exploration and validation in subsequent research.

**Systematic review registration:**

CRD42023465176, https://www.crd.york.ac.uk/PROSPERO/home.

## Introduction

OSAS is a clinical syndrome characterized by recurrent upper respiratory tract obstruction during sleep due to various causes, which leads to fragmented sleep and intermittent hypoxia during sleep periods (1). Studies have found that in patients with hypertension, coronary artery disease, heart failure, pulmonary hypertension, atrial fibrillation, and stroke, the prevalence of OSAS is as high as 40 to 80% (2). Its characteristics include repeated partial or complete respiratory pauses due to upper respiratory tract obstruction, affecting ventilation during sleep (3). Obstructive sleep apnea is very common among patients with cardiovascular diseases and is associated with the incidence and prevalence of hypertension, arrhythmias, coronary heart disease, heart failure, and stroke (4). It was found that the number of individuals affected by OSAS remains high, with an estimated 1 billion people suffering from OSAS worldwide (5).

As of now, the diagnostic process for OSAS is quite laborious. The gold standard method for diagnosing OSAS requires a polysomnography (PSG) system, a hospital setting, technical personnel, and a specialist physician (6). PSG involves recording, analyzing, and simultaneously collecting changes in multiple physiological signals, typically including but not limited to ECG and respiratory signals (6). However, a PSG device involves nearly 60 electrodes, making it difficult for a patient to have a normal sleep experience with so many connecting cables. Therefore, it becomes especially important to employ a method that not only offers high diagnostic reliability but is also simpler and more comfortable for patients (7).

In recent years, AI has begun to emerge and develop rapidly. In 2022, the highest investment was directed toward the healthcare and medical care sectors (8). Many anticipate that AI will achieve similar successes in the health sector, especially in diagnostics. Some believe that AI applications might even replace entire medical disciplines or create new roles to assist physicians (9). Against this background, medical image-based DL methods have gradually garnered extensive attention from researchers in clinical practice. For instance, in oncology, DL methods are employed to the identification of the benign or malignant nature of tumors based on pathological images, including early cancer detection, diagnosis, tumor classification and grading, molecular characterization, prognosis prediction, treatment response prediction, personalized treatment, automated radiation therapy workflows, discovery of novel anticancer drugs, and support in clinical trials (10, 11). Furthermore, we have noted other studies on DL and its application in diagnosing various types of diseases (12, 13).

In this context, some researchers have attempted to develop ECG-based DL models for detecting OSAS. However, the results from these DL approaches remain controversial, presenting certain challenges for the development of AI in this field. Therefore, this systematic review and meta-analysis was carried out to investigate the effectiveness and safety of DL methods based on medical images for real-time monitoring of OSAS, and to provide evidence-based opinions for the development and update of future real-time monitoring tools, such as wearable devices.

## Methods

### Study registration

The systematic review and meta-analysis adhered to the PRISMA, PRISMA NMA, and DMA guidelines, and prospectively registered on PROSPERO (ID: CRD42023465176).

### Eligibility criteria

#### Inclusion criteria

1. Studies such as case–control, cohort, case-cohort, nested case–control, and cross-sectional studies.
2. Studies that have fully constructed DL models for diagnosing OSA.
3. Some studies did not use other datasets for validation of the constructed models, and only performed cross-validation. These studies were also included in this systematic review.
4. Different DL studies were based on the same dataset. These studies were also included in our systematic review.
5. Included studies were original research reported in English.

#### Exclusion criteria

1. Studies such as meta-analyses, literature reviews, conference papers, guidelines, and expert opinions.
2. Given that this is a study on DL, which places a greater emphasis on the performance in the validation set, studies that did not perform any form of validation were excluded from our research.
3. Studies lacking an assessment of DL model accuracy with the following outcome measures: ROC curve, C-index, sensitivity, specificity, accuracy, precision, confusion matrix, F1 score, and calibration curve.
4. Studies focused on image segmentation.
5. Studies with populations being neonates and children.

### Data sources and search strategy

A systematical search was done for relevant literature published until September 25, 2025, in PubMed, Embase, Cochrane, and Web of Science databases, using MeSH + free-text terms, without limiting publication region or year (Table S1 for search strategy). To minimize the risk of missing newly published literature, we conducted a supplementary search of the databases on September 25, 2025.

### Study selection and data extraction

Obtained articles were imported into EndNote. After excluding duplicates, original studies that initially met the criteria were reviewed by titles and abstracts. Full texts of preliminary eligible articles were downloaded for further screening to select the final original studies for this review.

Prior to extracting data, a standardized data extraction spreadsheet was developed, including title, the first author, year of publication, author’s country, study type, patient source, image source, number of patients with sleep apnea syndrome, the total number of cases involved in the generation of the validation set, number of cases of sleep apnea syndrome in the validation set, model type used, and whether a comparison with clinical physicians was made.

The literature selection and data extraction were independently done by two researchers (Alimila Saiyitijiang; Zhihui Nai) and cross-checked. In the event of any disputes, a third reviewer was engaged for resolution.

### Risk of bias in studies

The risk of bias and applicability of the included studies were evaluated by adopting the QUADAS-2 tool (14). This tool evaluates four domains: patient selection, index test, reference standard, and flow and timing. Each domain contains specific questions answered as “Yes,” “No,” or “Uncertain,” corresponding to a judgment of bias risk as “Low,” “High,” or “Uncertain,” respectively. Studies are considered at low risk of bias if all signaling questions in each domain are answered with “Yes.” Any “No” among the answers to the signaling questions indicates potential bias, and the evaluator must then judge the risk of bias according to the guidelines provided. An “Uncertain” rating refers to situations with insufficient information for a definitive judgment.

### Synthesis methods

Data analysis was performed utilizing Stata 15.0. A bivariate mixed-effects model was employed for the meta-analysis. Pooled estimates of sensitivity, specificity, Positive likelihood ratio (PLR), Negative likelihood ratio (NLR), Diagnostic odds ratio (DOR), and corresponding 95% CIs, were calculated using this model. The area under the SROC curve was also estimated. Deek’s funnel plot was adopted to assess publication bias. Additionally, a nomogram was employed to evaluate the clinical applicability of DL. Throughout the analysis, the prevalence of OSA in the studies included was used as the prior probability. Furthermore, we conducted subgroup analyses on the methods of validation set generation (independent validation and k-fold cross-validation). *p* < 0.05 was deemed statistically significant.

## Results

### Study selection

**Figure 1:**
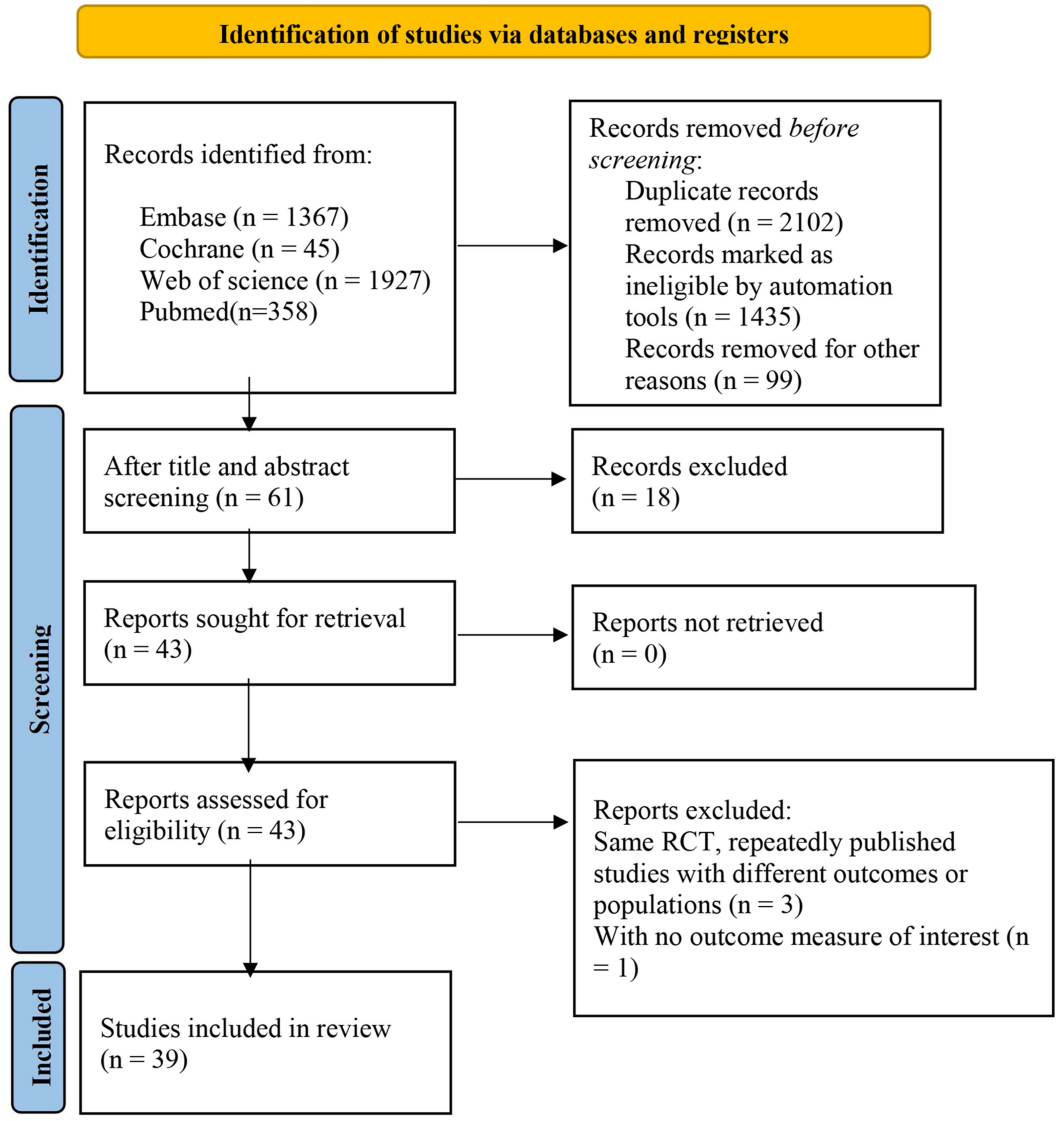
Literature screening flowchart.

From the various databases, we retrieved 3697 publications. After removing 2,102 duplicates, we further excluded articles based on the following criteria: not related to the subject of this study or not involving DL (1,435 articles), not written in English (18 articles), systematic reviews and guidelines as well as registries and those using other research methods (99 articles). Additionally, 4 articles were excluded due to being unable to download or incomplete data. Ultimately, 39 publications (15–53) were included in the study (Figure 1).

### Study characteristics

These articles were published primarily between 2018 and 2023, and only one (48) was published in 2011; 21 of these articles were from China (16, 17, 20–23, 25, 26, 29, 32–34, 36–39, 44–46, 50, 53), 5 from India (27, 29–31, 52), 3 from the United States (24, 40, 51), 3 from Iran (15, 19, 35), 2 from Korea (18, 41), 1 from Australia (42), 1 from Bangladesh (43), 1 from Jordan (48), and 1 from Turkey (49). Data from these articles were derived primarily from the 2000 Cardiology Association Physical Apnea-ECG Database, the PhysioNet publicly available Apnea-ECG Database, the UCDDB, and the Philips University Physical Apnea-ECG Database. In these studies, only 2 articles (33, 53) used 5-min segments, 1 (28) used 3-min segments, and 1 (26) used 6-min segments, while the rest of the articles used 1-min segments. Fourteen articles (15, 16, 22, 26, 28, 29, 31–33, 38, 44, 47–49) employed internal random sampling for validation, 13 (18, 19, 23, 24, 27, 30, 35, 36, 39, 40, 50–52) used K-fold cross-validation, and 13 (17, 19, 21, 25, 34, 37, 41–43, 45, 46, 51, 52) utilized external multicenter validation (Table 1).

**Table 1:** Essential details of the included literature.

No.	First author	Year of publication	Author’s country	Patient source	Image source	Segment duration (min)	Type of deep learning models	Total cases of OSA	Total number of cases
1	Bahrami, Mahsa	2022	Iran	Single-center	Single lead	1	CNN	P:19 I: 13066	P:32 I:34428
2	Chang, H. Y	2020	China, Taiwan	Single-center	Single lead	1	CNN	13,174	Segments: 34,2,131
3	Chen, J.	2022	China	Multicenter	Single lead	1	CNN		34,347 segments
4	Ullah, N	2023	Korea	Single-center	Single lead	1	DCDA	P: Unknown; I: 13,060	P: 70 I: 33,060
5	Zarei, A	2022	Iran	Multicenter	Single lead/dynamic 3-lead	1	CNN	Holdout set OSA segments: 6,550	P: 70 Segments: 34,313
6	Yang, Q.	2022	China	Internal	Single lead	1	ResNet	Total apnea segments: 13,048	P: 32 individuals; Segments: 34,129
7	Wang, Z.	2022	China	Multicenter	Single lead	1	URNet.	Database A contained 12,963 OSA segments and database B contained 10,337 OSA segments, with 8,328 in the training set and 2,009 in the test set.	Database A contained a total of 33,645 segments and database B contained 27,488 training and testing segments, involving 62 individuals
8	Fei Teng	2022	China	Single-center	Single lead	1	DCNN	Unknown	70 recordings
9	Febryan Setiawan	2022	China, Taiwan	Internal	Single lead	1	CNN	20 recordings	35 recordings
10	Tanmoy Pau	2022	USA	Single-center	Single lead	1	DFNN	A total of 1,072 ECG signals for OSA, with 271 test sequences being OSA	Total ECG sequences: 2,606, with 652 sequences in the test set
11	Qin, H	2022	China	Multicenter	Single lead	1	DCNN	Training set OSA sequences: 15,637; test set OSA sequences: 13,023	The training set consisted of 105 single-lead ECG recordings from overnight sessions, totaling 52,004 RR sequences; the test set included 70 ECG recordings, totaling 33,992 RR sequences.
12	Shuaicong Hu	2022	China	Internal	Single lead	6	CNN	A total of 13,066 OSA cases, with the test set comprising 6,552 OSA segments	70 recordings, totaling 34,428 segments (with 17,303 segments in the test set)
13	Kapil Gupta	2022	India	Single-center	Single lead	1	CNN	The database contained 70 overnight ECG records, which included a total of 11,620 segments after segmentation.	70 recordings
14	Liu, H	2023	China	Single-center	Single lead	3	CNN	Unknown	70 recordings (totaling 34,313 segments, of which 17,045 were in the release set, and 17,268 were in the holdout set)
15	Kumar Tyagi, P	2023	India	Single-center	Single lead	1	CNN	A total of 13,174 segments, with 6,657 OSA cases in the training group and 6,517 OSA cases in the validation group	70 recordings, totaling 34,212 min, 16,979 min in the training group, and 17,233 min in the validation group
16	Kumar, Chandra Bhushan	2023	India	Internal	3-lead	1	CNN		70 (35 for the training set and 35 for the test set)
17	Hemrajani, P	2023	India	Single-center	Single lead	1	CNN		70 individuals, 70 recordings (35 for the holdout set and 35 for the released set) The holdout set was used to train the model, and the release set was used for validation.
18	Chen, X	2023	China	Single-center	Single lead	1	BAF-Net		70 individuals, 70 recordings (half for the training set and half for the validation set)
19	Xianhui Chen	2022	China	Single-center	Single lead	5	CNN	46 (23 patients each in the training set and test set)	70 recordings, 70 individuals (34,039 min segments, with 16,945 segments in the test set)
20	Keyan Cao	2022	China	Multicenter	Single lead	1	CNN	In database 1, the training set contained 6,538 OSA segments, and the validation set contained 6,490 OSA segments; database 2 contained 2,633 OSA segments.	Database 1 consisted of 70 ECG signals totaling 33,715 segments, with 35 recordings making up a holdout set of 16,833 segments and 35 recordings in the release set totaling 16,882 segments. The other database included 25 ECG signals totaling 10,217 segments.
21	Mahsa Bahrami	2022	Iran	Single-center	Single lead	1	CNN		32 individuals with 70 ECG records
22	Yuankai YU	2021	China	Single-center	Single lead	1	CNN		32 individuals with 70 ECG records
23	Kunyang Li	2018	China	Multicenter	Single lead	1	CNN		Among the 70 recordings, the release set was utilized for training classifiers, while the holdout set was used for validation. In total, there were 34,313 segments included in the two groups, with the release set comprising 17,045 segments and the holdout group comprising 17,268 segments.
24	Lei Wang	2019	China	Single-center	Single lead	1	CNN		35 individuals, 35 recordings totaling 16,988 min of segments, with apnea types including 6,496 min and non-apnea types including 10,492 min.
25	Feng, K. C.	2021	China	Single-center	Single lead	1	CNN		32 individuals, 70 overnight ECG records (4 individuals each with 1 recording, 22 individuals each with 2 recordings, 2 individuals each with 3 recordings, and 4 individuals each with 4 recordings). The dataset was evenly divided into a release set and a holdout set.
26	Faust, O	2021	USA	Single-center	Single lead	1	CNN		35 recordings
27	Urtnasan, E.	2018	South Korea	Multicenter	Single lead	1	CNN	The training and testing datasets consisted of data from events involving 63 patients (34,281 events) and 19 patients (8,571 events), respectively.	A total of 82 subjects were randomized into two groups to form the training and testing datasets. The training dataset group included 17 cases of mild OSA, 23 cases of moderate OSA, and 23 cases of severe OSA. The testing dataset group comprised 5 cases of mild OSA, 7 cases of moderate OSA, and 7 cases of severe OSA.
28	Sharan, R. V	2020	Australia	Multicenter	Single lead	1	CNN		70 overnight ECG recordings, 35 recordings were used for training the model, and the other 35 were designated for testing
29	Mashrur, F. R	2021	Bangladesh	Multicenter	Single lead	1	CNN		70 subjects, 35 in the release set, while another 35 retained. The release dataset included 6,514 min of apnea events and 10,496 min of non-apnea events.
30	Junming Zhang	2021	China	Single-center	Single lead	1	CNN		70 recordings
31	Fang, H	2022	China	Multicenter	Single lead	1	CNN		32 subjects, a total of 33,752 segments were retained, with 16,743 segments allocated to the training set and 17,009 segments designated for the testing set.
32	Shen, Q	2021	China	Multicenter	Single lead	1	CNN		70 overnight ECG recordings, 35 recordings were used for training the model, and the other 35 were designated for testing
33	Thompson, S	2020	UK	Single-center	Single lead	1	CNN		70 individuals, 35 recordings, totaling 17,125 min (or 285 h and 25 min) of sleep time; of which 6,514 min (or 108 h and 34 min) were apnea, and 10,611 min (or 176 h and 51 min) were non-apnea.
34	Lweesy, K	2011	Jordan	Single-center	12-lead	1	CNN		25 individuals (1,500 data columns)
35	Nasifoglu, H	2021	Turkey	Single-center	Single lead		CNN		152 recordings
36	Niroshana, S. M. I.	2021	China, Taiwan	Single-center	Single lead	1	CNN		70 recordings, divided into two groups (release group and holdout group), each with 35 subjects
37	Sheta, A.	2021	USA	Single-center	Single lead		CNN		70 primary records, evenly divided into a learning set and a test set containing 35 records
38	Singh, H	2020	India	Multicenter	Single lead	1	CNN	From the Apnea-ECG database, a total of 6,509 cases of apnea events and 10,442 instances of normal events were obtained.	70 recordings
39	Wang, T	2019	China	Multicenter	Single lead	5	CNN		70 recordings

Validation set generation method	Number of OSA cases in validation set	Total cases in validation set
Internal validation		
Internal validation	6,517 min	Contents in the holdout set were all used for validation (17,234 min)
External validation		
10-fold cross validation	2,351 images (20% of the validation set)	5,951 images
External validation (multicenter)		35 recordings
External validation	17,164 segments were used for the test set, among which 6,536 segments indicated apnea	17,164 segments were used for the test set, among which 6,536 segments indicated apnea
External validation (multicenter)	2009	10 individuals, involving a total of 4,574 segments
Internal validation (random sampling)		35 (a total of 70 records, divided into groups of 35 each; 70% of one group was used for training, and the remaining 30% for testing)
10-fold cross validation		
10-fold cross validation	The test set included 271 OSA sequences	The test set included a total of 652 sequences
External validation (all 105 recordings were used for training, and all 70 recordings were used for testing).	OSA sequence: 13,023	70 recordings with a total of 33,992 RR sequences
Internal validation	Training set (35 recordings) and test set (35 recordings), with a total of 17,125 marked segments for both the training and test sets (6,514 marked as OSA and 10,611 marked as normal), and 17,303 segments (6,552 marked OSA and 10,751 marked as normal)	
A 10-fold cross validation strategy was utilized to construct the model. The entire dataset was divided into 10 parts, with eight parts used for training, one part for validation, and the remaining for testing. This process was repeated 10 times, each time with changes to the testing, validation, and training data.	80% was used as the training set, 10% as the test set.	
Internal validation (80% of the release set was used for training, 20% for validation; the holdout set was used for testing)	The training and validation sets had a ratio of 8:2	
Internal validation	6,517 min	35 recordings, 17,233 min
10-fold cross validation		
Internal validation	Unknown	35 recordings
Internal validation	Specifically, the training set was further divided into training and validation sets with a stratification ratio of 70%:30%	
Internal validation	23	35 recordings
External validation (multicenter)	In dataset 1, the release set consisted of 16,833 segments, with 6,538 being OSA segments and 10,295 being normal segments. The holdout set comprised 16,882 RRIs segments, with 6,490 for OSA and 10,392 for normal segments. The release set was used for training classifiers, while the holdout set was utilized to validate the performance of OSA detection algorithms. In the UCDDB dataset 2, 10,217 segments were retained, including 2,633 OSA segments and 7,584 normal segments, all of which were used for model testing	
10-fold stratified cross-validation	10% of the training data was used as a validation set	
10-fold cross validation		
Internal validation		
Internal validation		5 recordings
10-fold cross validation	The holdout set was used for testing, where OSA segments numbered 6,502, and normal segments 10,611	The total processed segments were 33,976 (release set: 16,863, holdout set: 17,113). The released collection was randomly divided into two parts: Part A (6,699 segments/14 subjects) and Part B (10,164 segments/21 subjects)
10-fold cross-validation + leave-one-out	Validation segments: 935,462	
External validation (multicenter)		
External validation (multicenter)	The training dataset comprised 16,817 segments, while the testing dataset contained 16,996 segments.	
External validation (multicenter)	The training, validation, and test sets included 11,367, 2,435, and 2,438 segments, respectively.	UCDDB dataset was subjected to training (7,025 segments), validation (1,505 segments), and testing (1,505 segments)
Internal validation	Training set (N210,680 + 130,050); validation set (N213,830 + 13,102)	
External validation (multicenter)		32 subjects, 70 ECG signal recordings, a total of 33,752 segments were retained. Of these, 16,743 segments were allocated to the training set, and 17,009 segments were designated for the testing set.
External validation (multicenter)	For the training sequence segments, there were 6,488 segments with OSA and 10,236 normal segments. The validation set retained a total of 16,988 sequence segments, comprising 6,489 OSA segments and 10,499 normal segments.	
Internal validation	Group A: OSA events: 6,250	Each experiment’s data were divided into 72% for training, 20% for testing, and 8% for validation
Internal validation		70% (1,052 data columns) were randomly selected for artificial neural network training, and the remaining 30% (448 data columns) were divided into two groups: one for validation (224 data columns) and the other for testing (224 data columns)
Internal validation		
10-fold cross validation		10-fold cross validation
10-fold cross validation	After preprocessing steps on the training set, the generated dataset contained 14,775 samples, of which 10,078 were marked as normal, and 4,679 were marked as affected by OSA	The test data generated after 10-fold cross-validation contained 4,935 samples, with 3,197 being marked as normal, and 1,738 marked as affected by OSA
10 fold cross validation		
External validation (multicenter)		

### Risk of bias in studies

**Figure 2:**
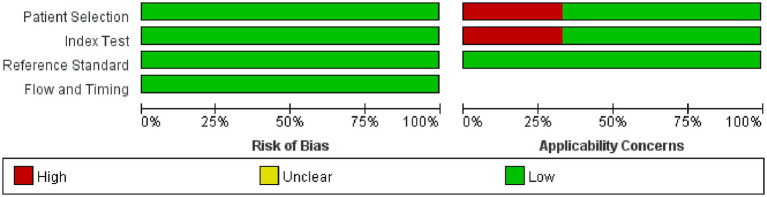
Methodological quality graph.

**Figure 3:**
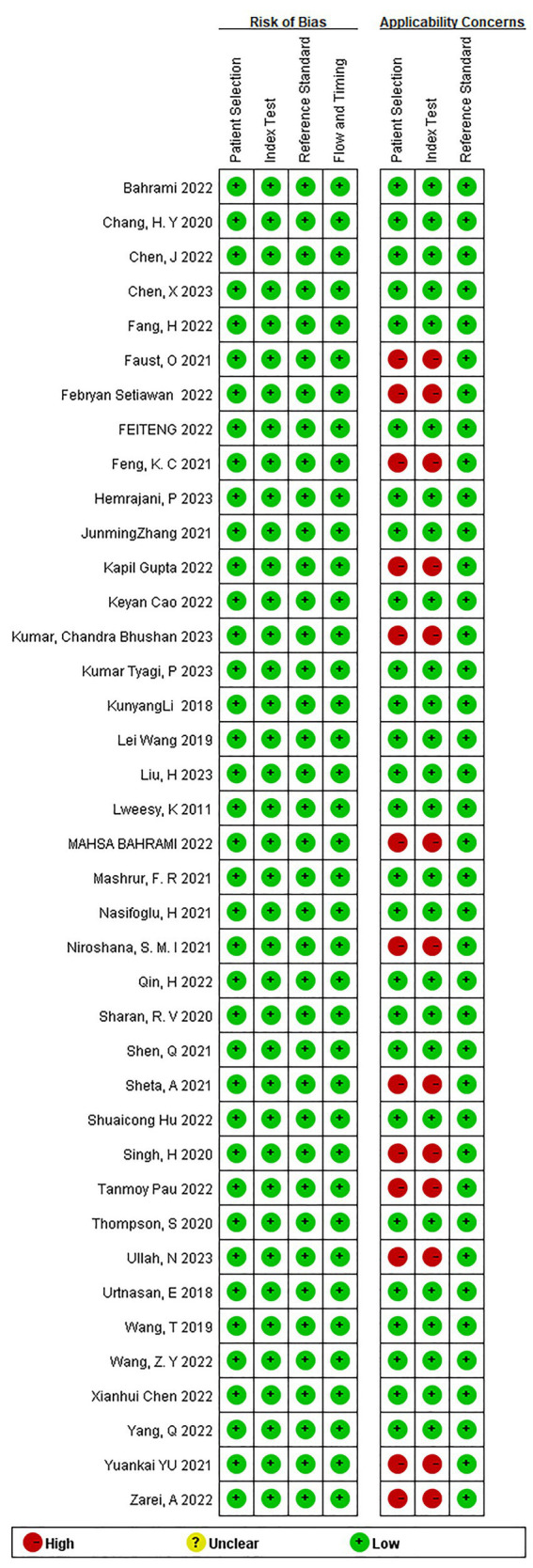
Methodological quality summary.

Utilizing the QUADAS-2 tool, we primarily assessed the overall risk of bias and concerns regarding applicability. In terms of overall bias risk, regarding case selection, none of the studies avoided case–control designs. However, since these studies primarily utilized ECG-based DL, where the modeling variables do not involve manual coding, we believed this introduced only a minimal risk of bias. Moreover, we considered all exclusions of cases to be appropriate; thus, from the perspective of case selection, those studies were judged to have a low risk of bias. As for the index test, most studies did not describe or provide information on whether the interpretation of outcomes was done without knowledge of the reference standard results, and there was no specific threshold established. Since both factors are unlikely to affect the results of DL, we considered the risk of bias from the index test perspective as low. All included studies were able to accurately distinguish the target disease states. The use of blinding in interpreting the reference standard results was not mentioned, but considering its minimal impact on DL models, we viewed the implementation and interpretation of the reference standard as having a low risk of bias. Most studies did not explain the time interval between the index test and the reference standard, but given that OSAS is a chronic condition, we assessed this as having a low risk of bias. Since all patients underwent the same reference standard and all cases meeting the inclusion criteria were analyzed, we considered the risk of bias in terms of the flow and timing of patients as low. Since the relevant included patients and backgrounds were well matched with the evaluation questions, and the reference standard is highly applicable, we assessed the applicability concerns related to patient selection and the reference standard as low. For independent validation sets, the index test’s implementation and interpretation matched the evaluation questions well, suggesting a low risk. However, for K-fold cross-validation, the implementation and interpretation of the index test presented a high risk of bias in matching the evaluation questions (Figures 2, 3).

## Meta-analysis

### Synthesized results

**Figure 4:**
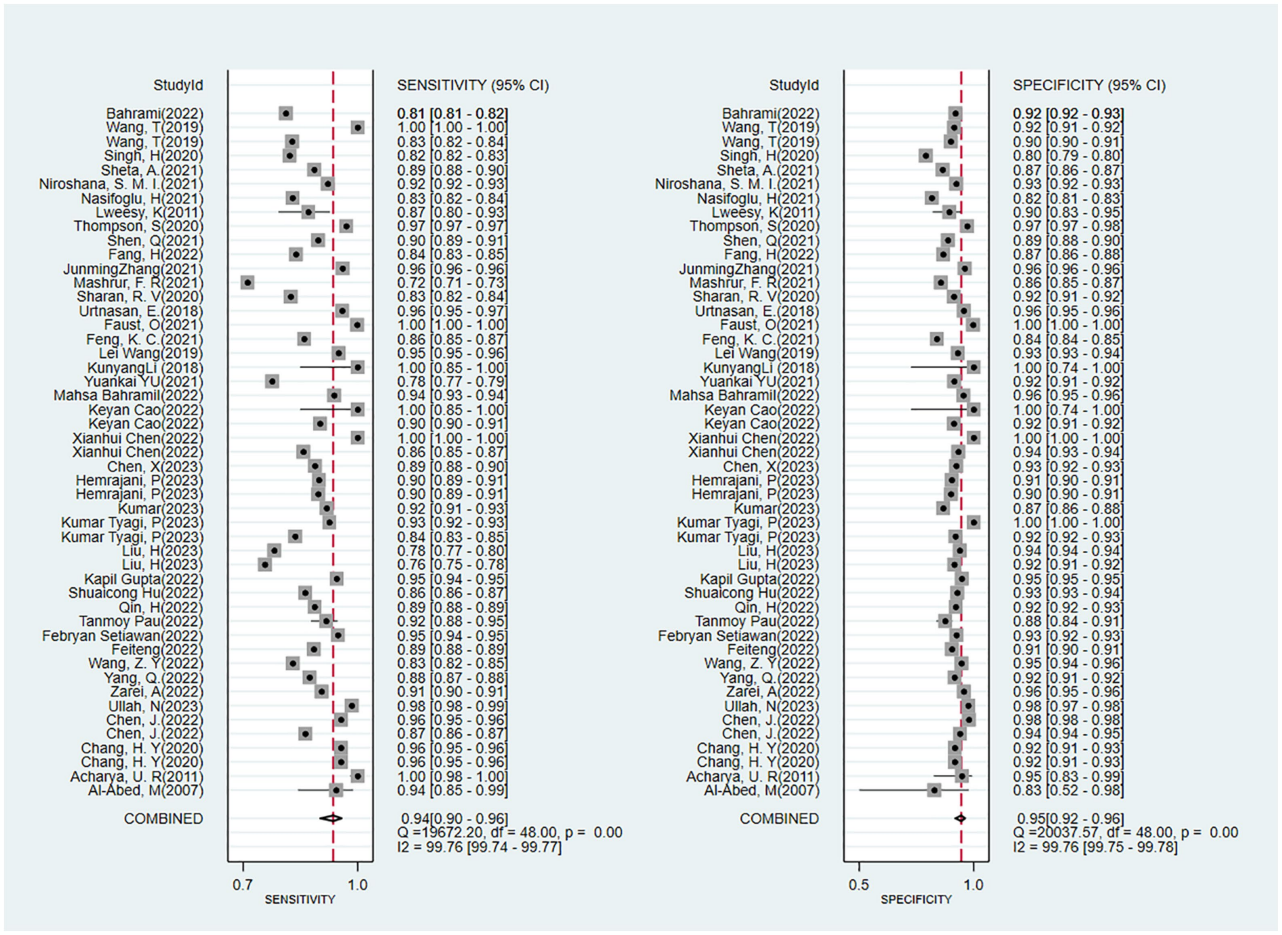
Forest plot of the meta-analysis results of the sensitivity and specificity of OSA detection by ECG segment-based DL models.

**Figure 5:**
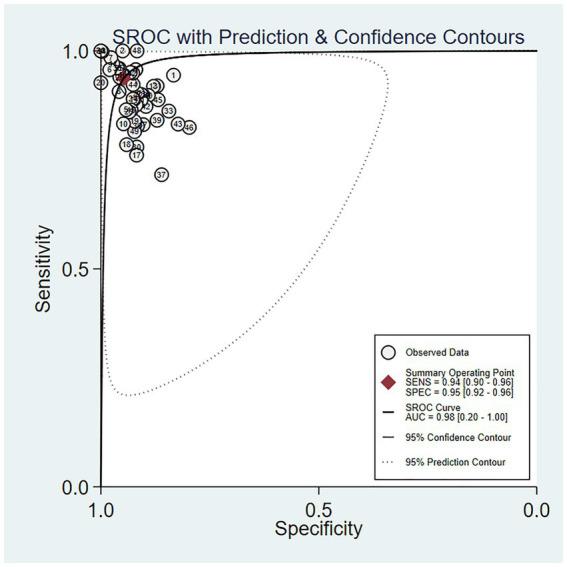
SROC curve of the meta-analysis results of OSA detection by ECG segment-based DL models.

The outcomes of our meta-analysis employing a bivariate mixed-effects model showed that the comprehensive validation set had a sensitivity of 0.93 (95% CI: 0.90–0.96), specificity of 0.95 (95% CI: 0.92–0.96), PLR of 17.7 (95% CI: 11.8–26.7), NLR of 0.07 (95% CI: 0.05–0.11), DOR of 252 (95% CI: 116–549), *I*^2^ = 99.76 (99.74–99.77) and an area under the SROC curve of 0.98 (95% CI: 0.42–1.00) (Figures 4, 5).

**Figure 6:**
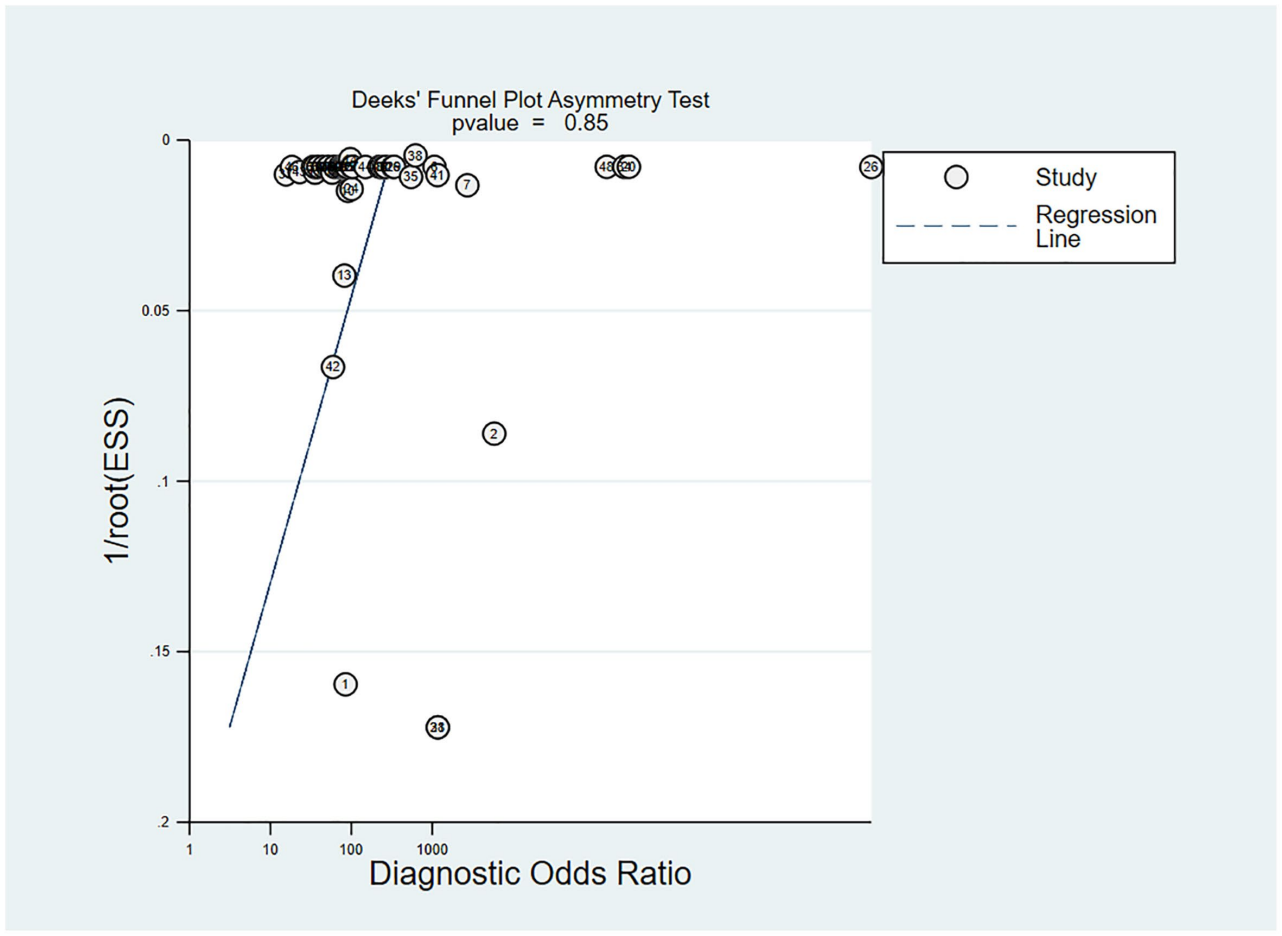
Nomogram of the meta-analysis results of OSA detection by ECG segment-based DL models.

**Figure 7:**
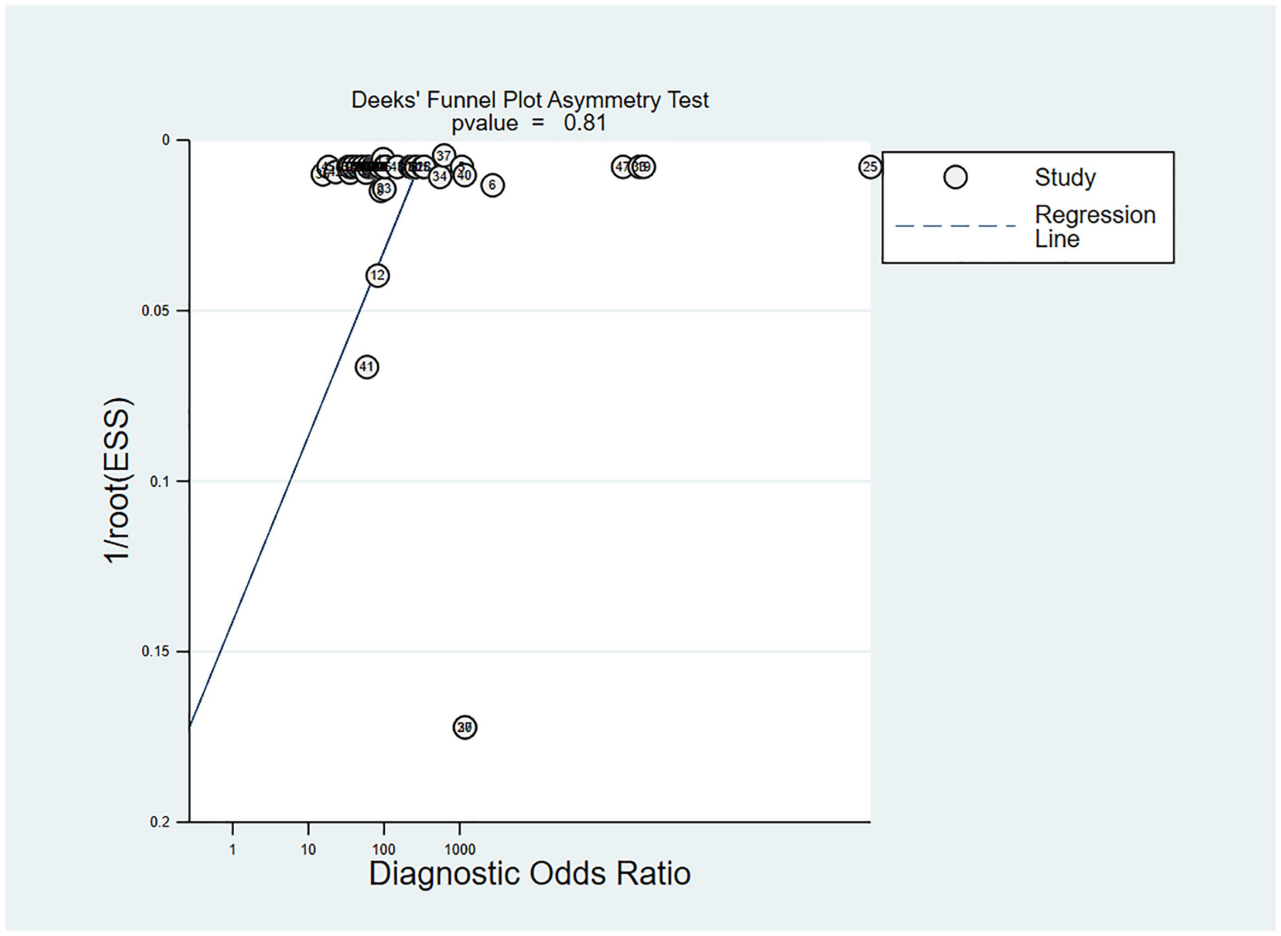
Deek’s funnel plot of meta-analysis results of OSA detection by ECG segment-based DL models.

In the studies included, about 30% of the ECG image segments were associated with OSAS. Using this data as a hypothesis for the prior probability of OSAS, when the DL judgment result was positive, the probability of the true result for sleep apnea syndrome was 88%; when the DL judgment result was negative, the probability of the true result not being sleep apnea syndrome was 97% (Figure 6). Deek’s funnel plot indicated no significant publication bias across the studies (Figure 7).

### Subgroup analysis

The sensitivity, specificity, PLR, NLR, DOR and SROC curve of the independent validation set were 0.93 (95% CI: 0.88–0.96), 0.95 (95% CI: 0.92–0.97), 19.5 (95% CI: 11.3–33.7), 0.07 (95% CI: 0.04–0.12), 274 (95% CI: 101–743), and 0.98 (95% CI: 0.42–1.00), respectively (Supplementary Figures S1, S2) and *I*^2^ = 99.76 (95% CI: 99.74–99.77). When the DL results were positive, the probability that the true result for apnea syndrome was 88%. When the DL judgment result was negative, the probability of the true result not being sleep apnea syndrome was 97% (Supplementary Figure S3). Deek’s funnel plot indicated no significant publication bias across the studies (Supplementary Figure S4).

The sensitivity, specificity, PLR, NLR, DOR and SROC curve of the K-fold cross-validation set were 0.94 (95% CI: 0.88–0.97), 0.94 (95% CI: 0.89–0.96), 15.0 (95% CI: 8.1–27.6), 0.07 (95% CI: 0.03–0.13), 227 (95% CI: 64–808), and 0.98 (95% CI: 0.65–1.00), I^2^ = 99.76 (99.74–99.77) respectively (Supplementary Figures S5, S6). When the DL results were positive, the probability that the true result for apnea syndrome was 90%. When the DL judgment result was negative, the probability of the true result not being sleep apnea syndrome was 96% (Supplementary Figure S7). Deek’s funnel plot revealed no significant publication bias across the studies (Supplementary Figure S8).

## Discussion

In this systematic review and meta-analysis, 39 original studies were analyzed using a bivariate mixed-effects model, synthesizing solely the results of the meta-analysis of the validation sets. From the analysis, it was observed that the sensitivity and specificity for real-time OSAS detection using ECG image-based DL were 0.93 (95% CI: 0.90–0.96) and 0.95 (95% CI: 0.92–0.96), respectively. For the independent validation sets, the sensitivity and specificity of detecting OSAS with ECG image-based DL were 0.93 (95% CI: 0.88–0.96) and 0.95 (95% CI: 0.92–0.97), respectively. For the K-fold cross-validation sets, the sensitivity and specificity of detecting OSAS using ECG image-based DL were 0.94 (95% CI: 0.88–0.97) and 0.94 (95% CI: 0.89–0.96), respectively.

In this study, we also noticed that some researchers have concentrated on the detection of OSAS using other methods such as polysomnography (PSG), biomarkers, imaging, etc. For instance, the method of “balloon angiography” used by Huysmans et al. (54) involved installing motion sensors under the bed of sleeping individuals to record coarse body movements, respiratory-related movements, and even cardiac motion during PSG. The combination of these three signals can largely provide a relatively better assessment of sleep state margins and sleep-related breathing disorders. However, this detection method is more complex and requires a higher level of environmental monitoring. However, the study results showed a screening sensitivity of 0.77 and specificity of 0.62 for patients with severe apneas, and for general apnea patients, a screening sensitivity of 0.72 and specificity of 0.70, both lower than those found in our study.

A study conducted by Zorlu D et al. (55) investigated the use of complete blood count parameters to predict the OSAS diagnosis and to grade the severity of OSAS. The study found that a lymphocyte value of 737.14 as a cutoff point showed a sensitivity (90.7%) and specificity (92.6%), indicating that the lymphocyte variable possesses a certain diagnostic value for mild OSAS. No significant cutoff values could be identified for moderate and severe OSAS groups, as the area under the ROC curve was not significant (*p* > 0.05) for these groups. Blood cell data are influenced by various factors, although eliminating the impact of patient-related factors on NLR, PLR, and WMR parameters was a notable strength in their study, they did not evaluate the impact of comorbidities, a history of corticosteroid treatments, or other inflammatory factors like calcitonin on complete blood count parameters.

Mahesh N et al. (56) also conducted a meta-analysis on diagnosing OSAS using the STOP-Bang questionnaire validated with polysomnography. In the sleep clinic population, the sensitivities for detecting any OSAS (AHI > 5), moderate to severe OSAS (AHI > 15), as well as severe OSAS (AHI > 30) were 90, 94, and 96%, respectively. Similarly, this pattern was noted in the surgical patient population. As the STOP-Bang score escalates, the likelihood of moderate and severe OSAS occurrence rises correspondingly. However, this study demonstrated moderate to high heterogeneity in the systematic review and meta-analysis, one factor of which was the variability of target populations in various studies and potential differences in the prevalence of OSAS among diverse populations. Additionally, there is a dearth of confirmatory studies in surgical patients. Moreover, it failed to achieve real-time detection, and the detection process was time-consuming.

In our study, we found that ECG-based methods for diagnosing OSA demonstrated highly favorable accuracy. ECG-based DL methods also achieved excellent diagnostic accuracy for OSA. However, significant challenges remain in the detection of OSA. For instance, OSAS is a sleep disorder characterized by repetitive cessation of airflow lasting at least 10 s per event, with each apnea episode accompanied by cardiovascular changes. During apnea, heart rate often decreases, and when breathing resumes, a relative tachycardia can be observed. Blood pressure drops during apnea episodes and rises at the end of apnea as sympathetic nerve activity increases. Oxygen saturation declines with the cessation of breathing, reaching its lowest point after several cycles of resumed breathing. The characteristics of fluctuations in blood oxygen saturation and heart rate are utilized by portable diagnostic devices for the early detection of sleep apnea (57).

Hypoxemia and hypercapnia episodes during sleep apnea can trigger physiological responses, including activation of the sympathetic nervous system, oxidative stress, inflammation, and endothelial dysfunction. Excessive daytime sleepiness may be described by patients as fatigue, low energy, or difficulty concentrating, reflecting an inability to maintain full wakefulness or alertness during the wakeful portion of the sleep–wake cycle (58). Studies have also indicated a certain association between OSA and traffic accidents. However, the relationship between OSA and traffic incidents is often complex and multifactorial in etiology, necessitating further investigation into the potential causes of these events. The process of screening for OSA solely through ECG measurements remains limited (59).

In our study of the ECG image-based DL models, the validation approach presented the true detection performance of AI for OSAS. In the included studies, 26 used an independent validation set to provide results, and 13 used K-fold cross-validation to provide results. According to the results, the sensitivity and specificity for real-time OSAS detection by ECG image-based DL were 0.93 (95% CI: 0.90–0.96) and 0.95 (95% CI: 0.92–0.96), respectively. For the independent validation sets, the sensitivity and specificity of detecting OSAS with ECG image-based DL were 0.93 (95% CI: 0.88–0.96) and 0.95 (95% CI: 0.92–0.97), respectively. For the K-fold cross-validation sets, the sensitivity and specificity of detecting OSAS using ECG image-based DL were, respectively, 0.94 (95% CI: 0.88–0.97) and 0.94 (95% CI: 0.89–0.96). The performance of independent validation sets and K-fold cross-validation showed no significant differences, indicating that DL methods for OSAS detection are highly stable. Moreover, among the included studies, Feiteng (22) designed a user-friendly OSAS monitoring system equipped with multimedia devices for accurate and efficient OSAS detection in intelligent healthcare management. In the study by Hemrajani, P (31), a compact, accurate, and portable wearable device, Sleepify, was developed to address the cumbersome and time-consuming nature of PSG, allowing patients to comfortably wear the device at home, record their ECG signals, and detect sleep apnea events, with the device alerting them to any incidents during the night.

### Advantages and limitations of the study

The DL models we investigated analyze long-duration single-lead ECG records of patients, and use one-dimensional ECG signals as input to detect apnea events, which also involve training and validation of the collected data. This approach does not involve QRS complex detection or analysis of RR intervals or cardiac function and requires less labor compared to traditional diagnostic methods, offering greater convenience and high accuracy.

In comparison with non-traditional detection methods, such as polysomnography (PSG) and blood biomarkers, our method is more straightforward in operation. Nevertheless, it is undeniable that our detection method is influenced by numerous factors. These factors encompass the instruments employed and the proficiency of the testing personnel. Additionally, given that the data utilized is sourced from public databases, there are indeed certain constraints in this respect.

As this is a retrospective study, it is seriously lacking in the explanation of blinding. In this deep learning, there are inherent rules for determining the threshold. We believe that it used the threshold rather than set the threshold probability. Therefore, we think that this does not bring an excessively high risk of bias to the entire training process.

Nevertheless, the specificity in our study population remains a challenge, mainly due to our modeling data primarily coming from four public databases: the 2000 Cardiology Association Physical Apnea-ECG Database, the PhysioNet publicly available Apnea-ECG Database, the UCDDB, and the Philips University Physical Apnea-ECG Database. These databases used different units for ECG recording, such as 1 min, 3 min (28), 5 min (33, 53), and 6 min ECG recording segments (26), the majority of them developed models based on segments with a duration of 1 min. Only a small number of models were constructed using segments of 5 min, 3 min, and 6 min. Moreover, some of these studies utilized cross - validation. As a result, it was challenging for us to perform further subgroup analysis. This also represents a limitation of our present study.

In addition, since the diagnosis of OSA was not clearly marked in most of the studies we included, we were unable to further present precise information about the diagnostic process in the original literature.

Additionally, the data included in our manuscript is not comprehensive; for instance, data from regions such as South America and New England are not represented. While there is a noted association between OSA and traffic accidents, the etiological relationship is often complex and multifactorial, requiring further investigation into the potential causes of these incidents. The screening process based solely on ECG measurements remains insufficient. Furthermore, some studies have highlighted the role of nocturnal blood oxygen saturation in the diagnosis of OSA. However, due to the limitations of the included literature, our study did not incorporate nocturnal blood pressure or nocturnal blood oxygen saturation measurements.

Also, We endeavored to utilize external validation to explore the influence of the same dataset across different studies on our research outcomes. Nevertheless, the scarcity of external validation datasets has impeded our ability to conduct a more in - depth analysis in this regard.

Among the 39 studies in our review, 33 used a 1-min window, one used a 3-min window, two used a 5-min window, one used a 6-min window, and two did not explicitly report the segment duration. For the meta-analysis, we employed a bivariate random-effects model, which requires at least four studies providing 2 × 2 contingency tables for diagnostic accuracy. Due to the limited number of studies in each subgroup defined by segment duration, we were unable to perform stratified subgroup analyses. This represents a limitation of our study.

Due to the existence of diverse frameworks in deep learning and the use of the bivariate mixed-effects model, at least four studies are required. Among the included studies, there are significant differences in these deep learning frameworks, and some have even conducted partial ablation experiments. Therefore, we were unable to further discuss the detection performance of different deep learning methods in this aspect. We directly summarized them as ideal diagnostic tools. This is also a major approach for discussing the detection of diseases by deep learning based on a single systematic review.

## Conclusion

The results from this study suggest that medical image-based DL methods have demonstrated marked efficacy and safety profiles in real-time monitoring of OSAS, and the use of AI for the diagnosis of OSAS is a novel and effective diagnostic approach, which can provide reliable evidence for future development and design of real-time monitoring tools for OSAS.

## Data Availability

The original contributions presented in the study are included in the article/Supplementary material, further inquiries can be directed to the corresponding author.
