# Supplementary material for: Accuracy of deep learning in diagnosis of apnea syndrome: a systematic review and meta-analysis
Source: Front Neurol. 2025 Dec 1;16:1663851. doi: 10.3389/fneur.2025.1663851 (PMC12702770; doi:10.3389/fneur.2025.1663851)
Supplement: Supplementary file 1 [file Supplementary_file_1.docx]

**Table S1:** Literature search strategy

**1.Pubmed**

| Search number | Query |
| --- | --- |
| #1 | "Sleep Apnea Syndromes"[Mesh] |
| #2 | "sleep apnea syndromes"[Title/Abstract] OR "sleep apnea syndrome"[Title/Abstract] OR "sleep hypopnea"[Title/Abstract] OR "sleep hypopneas"[Title/Abstract] OR "sleep apnea"[Title/Abstract] OR "sleep apneas"[Title/Abstract] OR "mixed sleep apnea"[Title/Abstract] OR "mixed sleep apneas"[Title/Abstract] OR "hypersomnia with periodic respiration"[Title/Abstract] OR "sleep disordered breathing"[Title/Abstract] OR "sleep disordered breathing"[Title/Abstract] OR "secondary central sleep apnea"[Title/Abstract] OR "primary central sleep apnea"[Title/Abstract] OR "central sleep apnea syndrome"[Title/Abstract] OR "central alveolar hypoventilation syndrome"[Title/Abstract] OR "central apnea"[Title/Abstract] OR "central apneas"[Title/Abstract] OR "obstructive sleep apneas"[Title/Abstract] OR "obstructive sleep apnea"[Title/Abstract] OR "obstructive sleep apnea syndrome"[Title/Abstract] OR "OSAHS"[Title/Abstract] OR (("Apnea"[MeSH Terms] OR "Apnea"[All Fields] OR "Apneas"[All Fields] OR "apnoeas"[All Fields] OR "apnoea"[All Fields]) AND "during sleep"[Title/Abstract]) OR "apnea syndrome"[Title/Abstract] OR "apnea syndromes"[Title/Abstract] OR (("Apnea"[MeSH Terms] OR "Apnea"[All Fields] OR "Apneas"[All Fields] OR "apnoeas"[All Fields] OR "apnoea"[All Fields]) AND "during sleep"[Title/Abstract]) OR "sleep apnea hypopnea"[Title/Abstract] OR "sleep related breathing abnormality"[Title/Abstract] |
| #3 | "Sleep Apnea Syndromes"[MeSH Terms] OR ("Sleep Apnea Syndromes"[Title/Abstract] OR "sleep apnea syndrome"[Title/Abstract] OR "sleep hypopnea"[Title/Abstract] OR "sleep hypopneas"[Title/Abstract] OR "sleep apnea"[Title/Abstract] OR "sleep apneas"[Title/Abstract] OR "mixed sleep apnea"[Title/Abstract] OR "mixed sleep apneas"[Title/Abstract] OR "hypersomnia with periodic respiration"[Title/Abstract] OR "sleep disordered breathing"[Title/Abstract] OR "sleep disordered breathing"[Title/Abstract] OR "secondary central sleep apnea"[Title/Abstract] OR "primary central sleep apnea"[Title/Abstract] OR "central sleep apnea syndrome"[Title/Abstract] OR "central alveolar hypoventilation syndrome"[Title/Abstract] OR "central apnea"[Title/Abstract] OR "central apneas"[Title/Abstract] OR "obstructive sleep apneas"[Title/Abstract] OR "obstructive sleep apnea"[Title/Abstract] OR "obstructive sleep apnea syndrome"[Title/Abstract] OR "OSAHS"[Title/Abstract] OR (("Apnea"[MeSH Terms] OR "Apnea"[All Fields] OR "Apneas"[All Fields] OR "apnoeas"[All Fields] OR "apnoea"[All Fields]) AND "during sleep"[Title/Abstract]) OR "apnea syndrome"[Title/Abstract] OR "apnea syndromes"[Title/Abstract] OR (("Apnea"[MeSH Terms] OR "Apnea"[All Fields] OR "Apneas"[All Fields] OR "apnoeas"[All Fields] OR "apnoea"[All Fields]) AND "during sleep"[Title/Abstract]) OR "sleep apnea hypopnea"[Title/Abstract] OR "sleep related breathing abnormality"[Title/Abstract]) |
| #4 | "Deep Learning"[Mesh] |
| #5 | "deep learning"[Title/Abstract] OR "transfer learning"[Title/Abstract] OR "ensemble learning"[Title/Abstract] OR "artificial intelligence"[Title/Abstract] OR "hierarchical learning"[Title/Abstract] OR "machine learning"[Title/Abstract] OR "neural networks"[Title/Abstract] OR "neural network"[Title/Abstract] OR "CNN"[Title/Abstract] OR "AlexNet"[Title/Abstract] OR "VGGNet"[Title/Abstract] OR "ResNet"[Title/Abstract] OR "GoogLeNet"[Title/Abstract] OR "VGG-11"[Title/Abstract] OR "VGG-13"[Title/Abstract] OR "VGG-16"[Title/Abstract] OR "VGG-19"[Title/Abstract] OR "VGG11"[Title/Abstract] OR "VGG13"[Title/Abstract] OR "VGG16"[Title/Abstract] OR "VGG19"[Title/Abstract] OR "ResNet50"[Title/Abstract] OR "ResNet101"[Title/Abstract] OR "ResNet34"[Title/Abstract] OR "ResNet18"[Title/Abstract] |
| #6 | "Deep Learning"[MeSH Terms] OR "Deep Learning"[Title/Abstract] OR "transfer learning"[Title/Abstract] OR "ensemble learning"[Title/Abstract] OR "artificial intelligence"[Title/Abstract] OR "hierarchical learning"[Title/Abstract] OR "machine learning"[Title/Abstract] OR "neural networks"[Title/Abstract] OR "neural network"[Title/Abstract] OR "CNN"[Title/Abstract] OR "AlexNet"[Title/Abstract] OR "VGGNet"[Title/Abstract] OR "ResNet"[Title/Abstract] OR "GoogLeNet"[Title/Abstract] OR "VGG-11"[Title/Abstract] OR "VGG-13"[Title/Abstract] OR "VGG-16"[Title/Abstract] OR "VGG-19"[Title/Abstract] OR "VGG11"[Title/Abstract] OR "VGG13"[Title/Abstract] OR "VGG16"[Title/Abstract] OR "VGG19"[Title/Abstract] OR "ResNet50"[Title/Abstract] OR "ResNet101"[Title/Abstract] OR "ResNet34"[Title/Abstract] OR "ResNet18"[Title/Abstract] |
| #7 | ("Sleep Apnea Syndromes"[MeSH Terms] OR ("Sleep Apnea Syndromes"[Title/Abstract] OR "sleep apnea syndrome"[Title/Abstract] OR "sleep hypopnea"[Title/Abstract] OR "sleep hypopneas"[Title/Abstract] OR "sleep apnea"[Title/Abstract] OR "sleep apneas"[Title/Abstract] OR "mixed sleep apnea"[Title/Abstract] OR "mixed sleep apneas"[Title/Abstract] OR "hypersomnia with periodic respiration"[Title/Abstract] OR "sleep disordered breathing"[Title/Abstract] OR "sleep disordered breathing"[Title/Abstract] OR "secondary central sleep apnea"[Title/Abstract] OR "primary central sleep apnea"[Title/Abstract] OR "central sleep apnea syndrome"[Title/Abstract] OR "central alveolar hypoventilation syndrome"[Title/Abstract] OR "central apnea"[Title/Abstract] OR "central apneas"[Title/Abstract] OR "obstructive sleep apneas"[Title/Abstract] OR "obstructive sleep apnea"[Title/Abstract] OR "obstructive sleep apnea syndrome"[Title/Abstract] OR "OSAHS"[Title/Abstract] OR (("Apnea"[MeSH Terms] OR "Apnea"[All Fields] OR "Apneas"[All Fields] OR "apnoeas"[All Fields] OR "apnoea"[All Fields]) AND "during sleep"[Title/Abstract]) OR "apnea syndrome"[Title/Abstract] OR "apnea syndromes"[Title/Abstract] OR (("Apnea"[MeSH Terms] OR "Apnea"[All Fields] OR "Apneas"[All Fields] OR "apnoeas"[All Fields] OR "apnoea"[All Fields]) AND "during sleep"[Title/Abstract]) OR "sleep apnea hypopnea"[Title/Abstract] OR "sleep related breathing abnormality"[Title/Abstract])) AND ("Deep Learning"[MeSH Terms] OR ("Deep Learning"[Title/Abstract] OR "transfer learning"[Title/Abstract] OR "ensemble learning"[Title/Abstract] OR "artificial intelligence"[Title/Abstract] OR "hierarchical learning"[Title/Abstract] OR "machine learning"[Title/Abstract] OR "neural networks"[Title/Abstract] OR "neural network"[Title/Abstract] OR "CNN"[Title/Abstract] OR "AlexNet"[Title/Abstract] OR "VGGNet"[Title/Abstract] OR "ResNet"[Title/Abstract] OR "GoogLeNet"[Title/Abstract] OR "VGG-11"[Title/Abstract] OR "VGG-13"[Title/Abstract] OR "VGG-16"[Title/Abstract] OR "VGG-19"[Title/Abstract] OR "VGG11"[Title/Abstract] OR "VGG13"[Title/Abstract] OR "VGG16"[Title/Abstract] OR "VGG19"[Title/Abstract] OR "ResNet50"[Title/Abstract] OR "ResNet101"[Title/Abstract] OR "ResNet34"[Title/Abstract] OR "ResNet18"[Title/Abstract])) |

**2.Cochrane**

| Search number | Query |
| --- | --- |
| #1 | MeSH descriptor: [Sleep Apnea Syndromes] explode all trees |
| #2 | (Sleep Apnea Syndromes):ti,ab,kw OR (Sleep Apnea Syndrome):ti,ab,kw OR (Sleep Hypopnea):ti,ab,kw OR (Sleep Hypopneas):ti,ab,kw OR (Sleep Apnea):ti,ab,kw |
| #3 | (Sleep Apneas):ti,ab,kw OR (Mixed Sleep Apnea):ti,ab,kw OR (Mixed Sleep Apneas):ti,ab,kw OR (Hypersomnia with Periodic Respiration):ti,ab,kw OR (Sleep-Disordered Breathing):ti,ab,kw |
| #4 | (Sleep Disordered Breathing):ti,ab,kw OR (Secondary Central Sleep Apnea):ti,ab,kw OR (Primary Central Sleep Apnea):ti,ab,kw OR (Central Sleep Apnea Syndrome):ti,ab,kw OR (Central Alveolar Hypoventilation Syndrome):ti,ab,kw |
| #5 | (Central Apnea):ti,ab,kw OR (Central Apneas):ti,ab,kw OR (Obstructive Sleep Apneas):ti,ab,kw OR (Obstructive Sleep Apnea):ti,ab,kw OR (Obstructive Sleep Apnea Syndrome):ti,ab,kw |
| #6 | (OSAHS):ti,ab,kw OR (apnea during sleep):ti,ab,kw OR (apnea syndrome):ti,ab,kw OR (apnea syndromes):ti,ab,kw OR (apneas during sleep):ti,ab,kw |
| #7 | (sleep apnea-hypopnea):ti,ab,kw OR (sleep related breathing abnormality):ti,ab,kw |
| #8 | #1 or #2 or #3 or #4 or #5 or #6 or #7 |
| #9 | MeSH descriptor: [Deep Learning] explode all trees |
| #10 | (deep learning):ti,ab,kw OR (Transfer Learning):ti,ab,kw OR (Ensemble Learning):ti,ab,kw OR (artificial intelligence):ti,ab,kw OR (Hierarchical Learning):ti,ab,kw |
| #11 | (Machine Learning):ti,ab,kw OR (Neural Networks):ti,ab,kw OR (Neural Network):ti,ab,kw OR (CNN):ti,ab,kw OR (AlexNet):ti,ab,kw |
| #12 | (VGGNet):ti,ab,kw OR (ResNet):ti,ab,kw OR (GoogLeNet):ti,ab,kw OR (VGG-11):ti,ab,kw OR (VGG-13):ti,ab,kw |
| #13 | (VGG-16):ti,ab,kw OR (VGG-19):ti,ab,kw OR (VGG11):ti,ab,kw OR (VGG13):ti,ab,kw OR (VGG16):ti,ab,kw |
| #14 | (VGG19):ti,ab,kw OR (ResNet50):ti,ab,kw OR (ResNet101):ti,ab,kw OR (ResNet34):ti,ab,kw OR (ResNet18):ti,ab,kw |
| #15 | #9 or #10 or #11 or #12 or #13 or #14 |
| #16 | #8 and #15 |

**3.Embase**

| Search number | Query |
| --- | --- |
| #1 | 'sleep apnea syndromes'/exp |
| #2 | sleep apnea syndromes'/exp OR 'sleep apnea syndromes' OR (('sleep'/exp OR sleep) AND ('apnea'/exp OR apnea) AND syndromes) OR 'sleep apnea syndrome':ab,ti OR 'sleep hypopnea':ab,ti OR 'sleep hypopneas':ab,ti OR 'sleep apnea':ab,ti OR 'sleep apneas':ab,ti OR 'mixed sleep apnea':ab,ti OR 'mixed sleep apneas':ab,ti OR 'hypersomnia with periodic respiration':ab,ti OR 'sleep-disordered breathing':ab,ti OR 'sleep disordered breathing':ab,ti OR 'secondary central sleep apnea':ab,ti OR 'primary central sleep apnea':ab,ti OR 'central sleep apnea syndrome':ab,ti OR 'central alveolar hypoventilation syndrome':ab,ti OR 'central apnea':ab,ti OR 'central apneas':ab,ti OR 'obstructive sleep apneas':ab,ti OR 'obstructive sleep apnea':ab,ti OR 'obstructive sleep apnea syndrome':ab,ti OR osahs:ab,ti OR 'apnea during sleep':ab,ti OR 'apnea syndrome':ab,ti OR 'apnea syndromes':ab,ti OR 'apneas during sleep':ab,ti OR 'sleep apnea-hypopnea':ab,ti OR 'sleep related breathing abnormality':ab,ti |
| #3 | #1 OR #2 |
| #4 | deep learning'/exp |
| #5 | 'deep learning'/exp OR 'deep learning' OR (deep AND ('learning'/exp OR learning)) OR 'transfer learning':ab,ti OR 'ensemble learning':ab,ti OR 'artificial intelligence':ab,ti OR 'hierarchical learning':ab,ti OR 'machine learning':ab,ti OR 'neural networks':ab,ti OR 'neural network':ab,ti OR cnn:ab,ti OR alexnet:ab,ti OR vggnet:ab,ti OR resnet:ab,ti OR googlenet:ab,ti OR 'vgg 11':ab,ti OR 'vgg 13':ab,ti OR 'vgg 16':ab,ti OR 'vgg 19':ab,ti OR vgg11:ab,ti OR vgg13:ab,ti OR vgg16:ab,ti OR vgg19:ab,ti OR resnet50:ab,ti OR resnet101:ab,ti OR resnet34:ab,ti OR resnet18:ab,ti |
| #6 | #4 OR #5 |
| #7 | #3 AND #6 |

**4.Web of science**

| Search number | Query |
| --- | --- |
| #1 | Sleep Apnea Syndromes (Topic) OR Sleep Apnea Syndrome (Topic) OR Sleep Hypopnea (Topic) OR Sleep Hypopneas (Topic) OR Sleep Apnea (Topic) OR Sleep Apneas (Topic) OR Mixed Sleep Apnea (Topic) OR Mixed Sleep Apneas (Topic) OR Hypersomnia with Periodic Respiration (Topic) OR Sleep-Disordered Breathing (Topic) OR Sleep Disordered Breathing (Topic) OR Secondary Central Sleep Apnea (Topic) OR Primary Central Sleep Apnea (Topic) OR Central Sleep Apnea Syndrome (Topic) OR Central Alveolar Hypoventilation Syndrome (Topic) OR Central Apnea (Topic) OR Central Apneas (Topic) OR Obstructive Sleep Apneas (Topic) OR Obstructive Sleep Apnea (Topic) OR Obstructive Sleep Apnea Syndrome (Topic) OR OSAHS (Topic) OR apnea during sleep (Topic) OR apnea syndrome (Topic) OR apnea syndromes (Topic) OR apneas during sleep (Topic) OR sleep apnea-hypopnea (Topic) OR sleep related breathing abnormality (Topic) |
| #2 | deep learning (Topic) OR Transfer Learning (Topic) OR Ensemble Learning (Topic) OR artificial intelligence (Topic) OR Hierarchical Learning (Topic) OR Machine Learning (Topic) OR Neural Networks (Topic) OR Neural Network (Topic) OR CNN (Topic) OR AlexNet (Topic) OR VGGNet (Topic) OR ResNet (Topic) OR GoogLeNet (Topic) OR VGG-11 (Topic) OR VGG-13 (Topic) OR VGG-16 (Topic) OR VGG-19 (Topic) OR VGG11 (Topic) OR VGG13 (Topic) OR VGG16 (Topic) OR VGG19 (Topic) OR ResNet50 (Topic) OR ResNet101 (Topic) OR ResNet34 (Topic) OR ResNet18 (Topic) |
| #3 | #1 AND #2 |

**
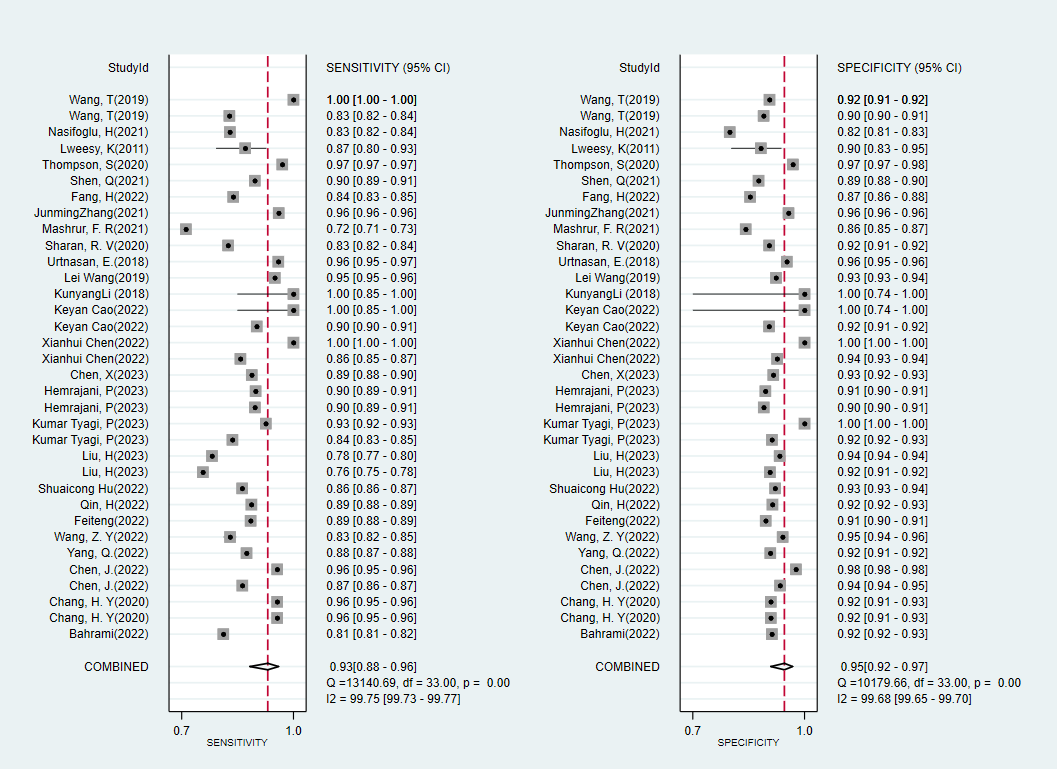
**

**Figure S1.** Forest plot of the meta-analysis results of the sensitivity and specificity of OSA detection by ECG segment-based DL models – independent validation set.


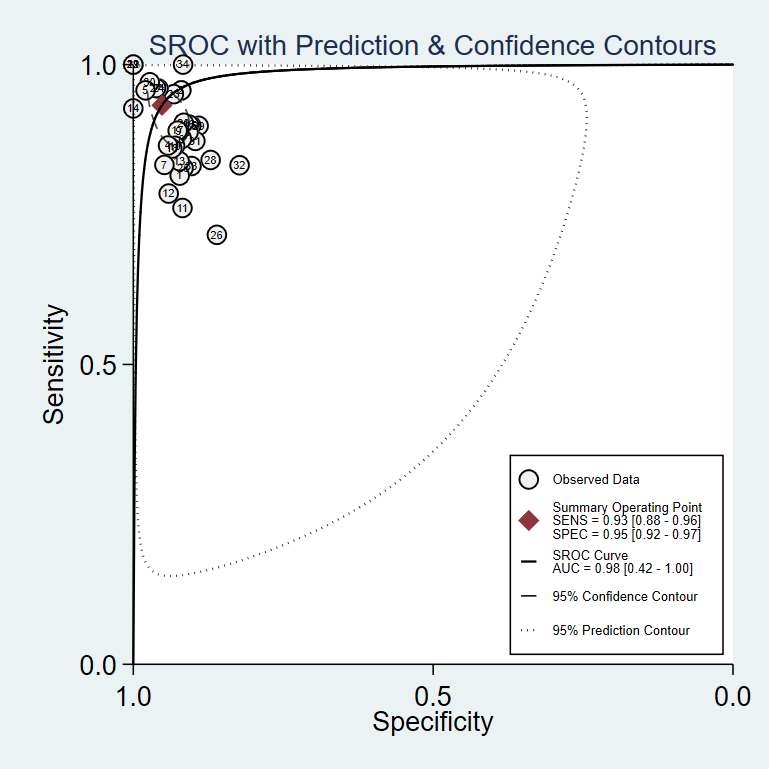


**Figure S2.** SROC curve of the meta-analysis results of OSA detection by ECG segment-based DL models – independent validation set.


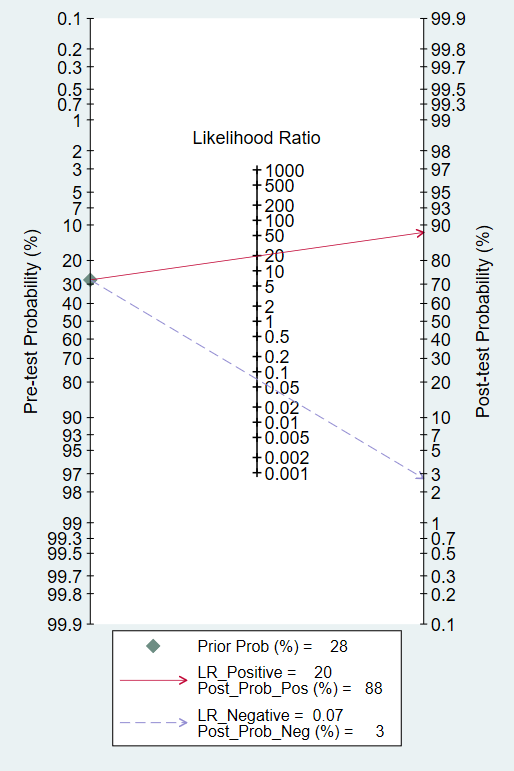


**Figure S3.** Nomogram of the meta-analysis results of OSA detection by ECG segment-based DL models – independent validation set.


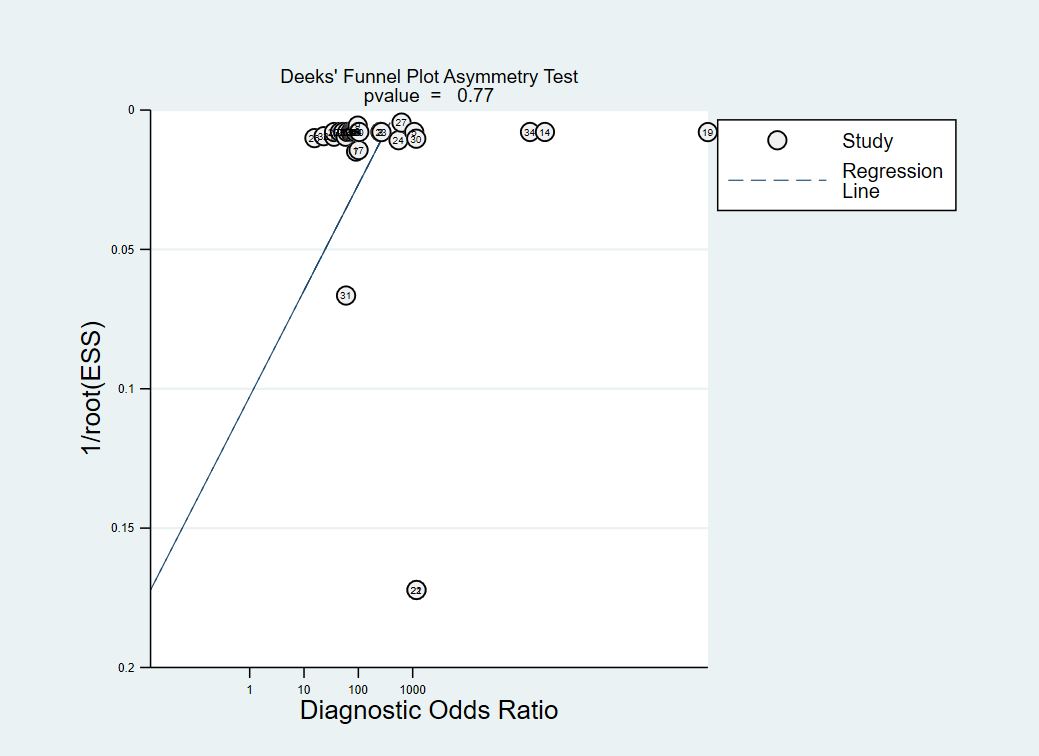


**Figure S4.** Deek's funnel plot of meta-analysis results of OSA detection by ECG segment-based DL models – independent validation set.


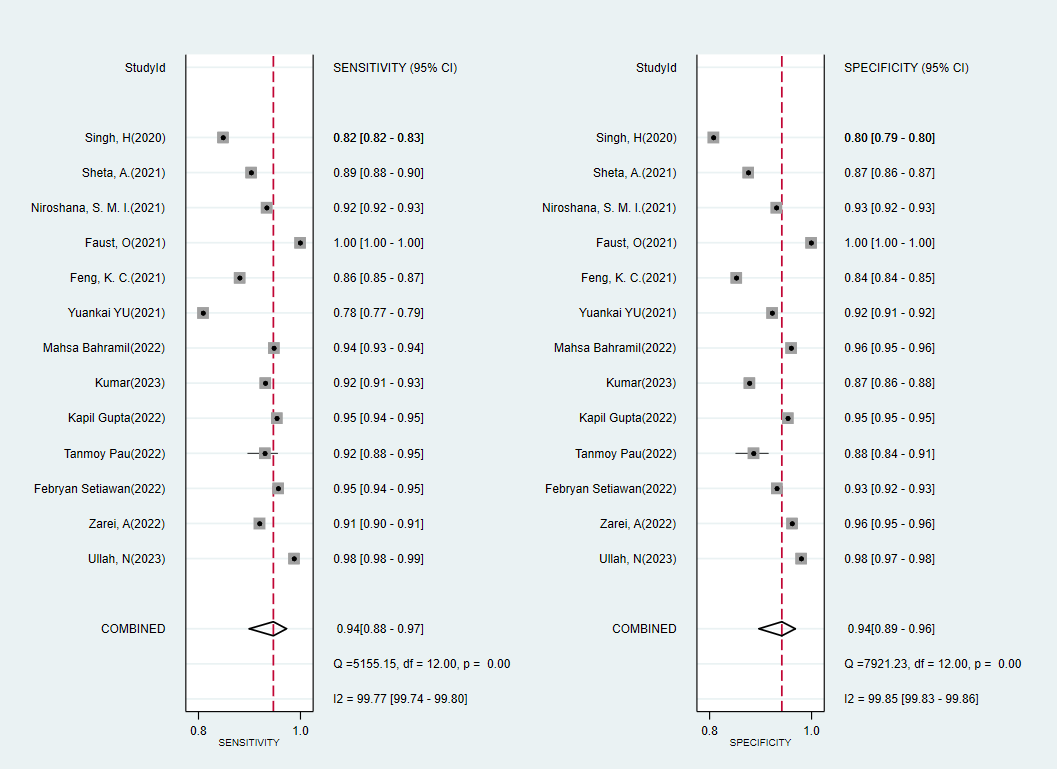


**Figure S5.** Forest plot of the meta-analysis results of the sensitivity and specificity of OSA detection by ECG segment-based DL models – K-fold cross-validation set.

**
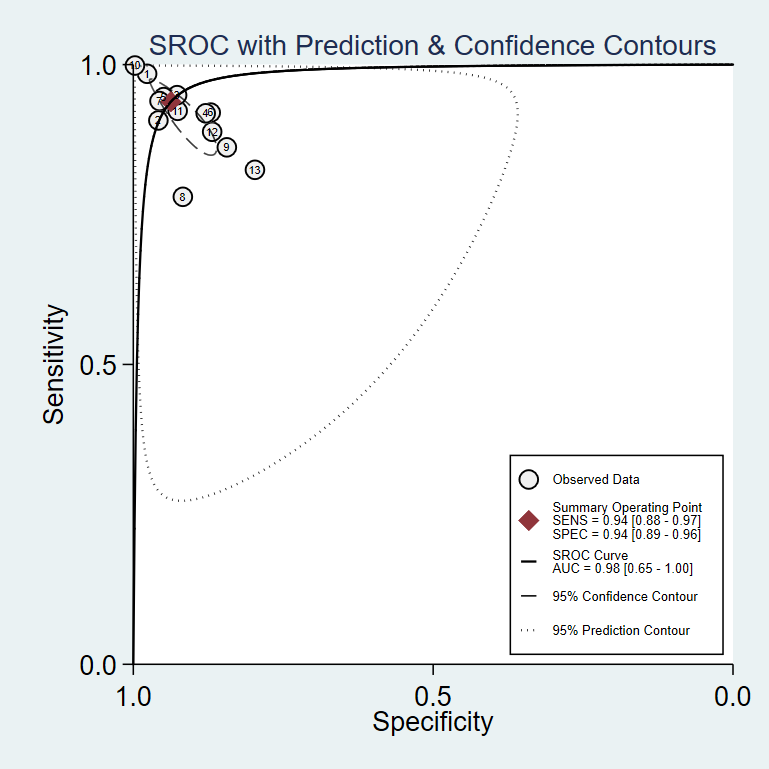
**

**Figure S6.** SROC curve of the meta-analysis results of OSA detection by ECG segment-based DL models – K-fold cross-validation set.


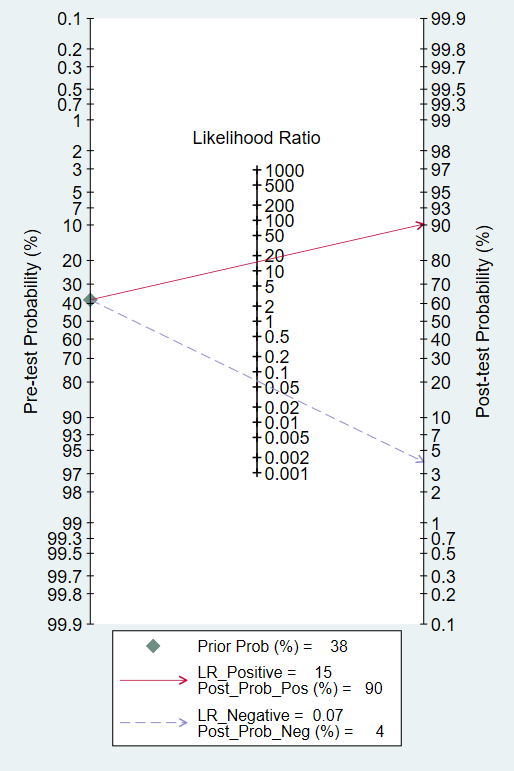


**Figure S7.** Nomogram of the meta-analysis results of OSA detection by ECG segment-based DL models – K-fold cross-validation set.


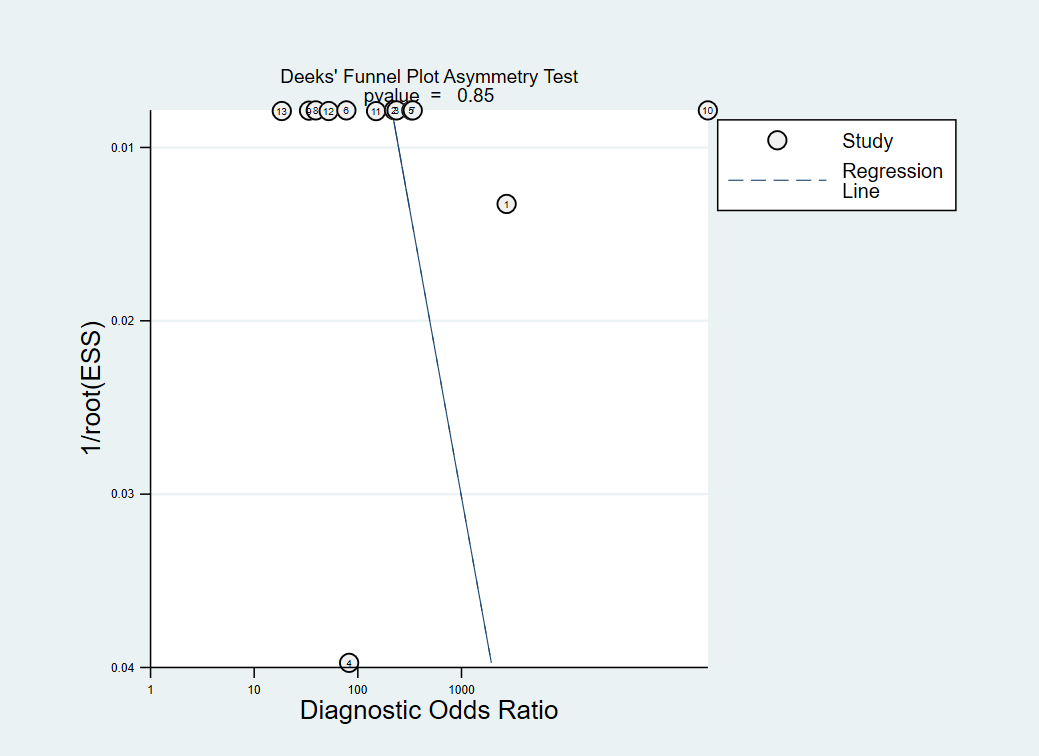


**Figure S8.** Deek's funnel plot of meta-analysis results of OSA detection by ECG segment-based DL models – K-fold cross-validation set.
